# Design, Synthesis and Biological Evaluation of Novel Piperazinone Derivatives as Cytotoxic Agents

**DOI:** 10.34172/apb.2020.051

**Published:** 2020-05-11

**Authors:** Saeed Ghasemi, Simin Sharifi, Javid Shahbazi Mojarrad

**Affiliations:** ^1^Department of Medicinal Chemistry, School of Pharmacy, Guilan University of Medical Sciences, Rasht, Iran.; ^2^Dental and Periodontal Research Center, Tabriz University of Medical Sciences, Tabriz, Iran.; ^3^Department of Medicinal Chemistry, Faculty of Pharmacy, Tabriz University of Medical Sciences, Tabriz, Iran.

**Keywords:** Anticancer activity, Bioisosteric replacement, Piperazinone, Chemical synthesis

## Abstract

***Purpose:*** In this study, a series of piperazin-2-one derivatives were prepared through bioisosteric substitution of the imidazole ring of L-778,123 (imidazole-containing FTase inhibitor) and rearrangement of groups based on the tipifarnib structure. Final compounds were evaluated for their cytotoxic activities on cancer and normal cell lines by MTT assay.

***Methods:*** Methyl α-bromophenylacetic acid and 1-(3-chlorophenyl) piperazin-2-one were synthesized using previously described methods. Methyl 2-(4-chlorophenyl)-2-(4-(3- chlorophenyl)-3-oxopiperazin-1-yl) acetate was prepared by reaction between these two compounds in presence of potassium carbonate. Finally, methoxy group of ester was substituted by various amines such as guanidine, thiourea, urea and hydrazide. The synthesized compounds were tested for their cytotoxicity against colon cancer (HT-29) and lung cancer (A549) cell lines as well as MRC-5 (normal fetal lung fibroblasts) cells as a healthy cell line using MTT colorimetric assay method.

***Results:*** Replacement of imidazole moiety with guanidine, thiourea, and hydrazide could increase cytotoxicity toward all three cell lines. Some substituents, such as amine, urea, and hydroxylamine exhibited significant cytotoxicity (<500 µM) but lower than L-778,123 as standard compound. Hydroxyl and methoxy substituents did not show significant cytotoxicity. Imidazole substituent group revealed cytotoxicity similar to L-778,123 All compounds showed lower cytotoxic activity against normal cell lines compared with cancer cell lines.

***Conclusion:*** It seems the electron density of substituted groups and rearrangement of groups may significantly increase cytotoxic activity

## Introduction

In developed countries, cancer has changed into one of the most important causes of death. Because of the complications of existing agents such as drug resistance and toxicities, the introduction of novel anticancer compounds is essential.^1,2+^

**Figure 1:**
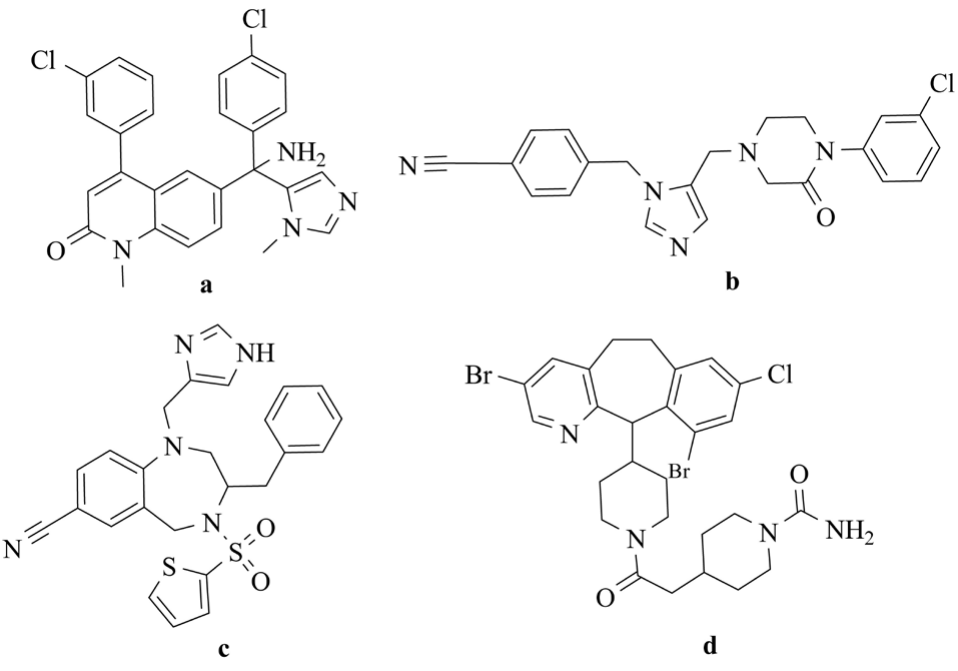
Structures of FTIs: (a) L-778,123 (b)Tipifarnib (c)BMS-214662 (d)Lonafarnib.

Studies have demonstrated that 30% of human cancers occur as a result of the mutation of ras genes.^3^ Ras proteins activate signal transduction pathways with an essential role in cell growth. The post-translational modification should be done for activation by several sequential enzymatic steps. Farnesylation of Ras protein can be interrupted by farnesyltransferase inhibitors (FTIs) and cause suppression of the tumor cells growth that depends on Ras.^4,5^ Thus, the research for the development of the cancer treatment by novel FTIs has recently attracted a great deal of attention. Several potent non-thiol FTIs such as lonafarnib, L-778,123, BMS-214662, and tipifarnib have been introduced which can be used for hematological cancers and solid tumors therapy (Figure 1).^5-9^

Imidazole ring has a crucial role in the interaction of these groups with the enzyme. Researchers have shown that although the imidazole substitution with other heterocyclic rings such as pyridine in FTIs can improve the cytotoxicity, it can decrease inhibitory activity on human farnesyltransferase. Besides, studies of cancerous cell lines have suggested that more than 70% of the cancer cells without mutation in Ras proteins can be sensitive to FTIs.^4,5^ These results revealed the existence of extra mechanisms apart from farnesyltransferase enzyme inhibition.^9-13^

Also, some groups such as semicarbazide, guanidine, thiourea, and urea derivatives exhibit potent cytotoxic activity with diverse mechanisms.^14-18^

In this research, the synthesis and cytotoxic activity profile of new 1-(3-chlorophenyl) 2-piperazinone compounds containing imidazole moiety and bioisosteres of imidazole such as guanidine, thiourea, etc. were reported. The study also explores the structure of compounds rearranged based on the tipifarnibe structure as potent FTIs. The novel compounds are investigated on normal human lung cells as well as two human cancerous cells including MRC-5 (normal fetal lung fibroblasts) and HT29 (human colonic adenocarcinoma cells) and A549 (adenocarcinoma human alveolar basal epithelial cells) cells.

## Materials and Methods

### Chemistry

Reagents and solvents were bought from Merck and Sigma Aldrich. An Electrothermal-9100 melting point apparatus was used for the melting point determination and are uncorrected. Shimadzu 4300 spectrophotometer (potassium bromide dicks) was used for recording the IR spectra. ^1^HNMR and ^13^CNMR spectra were acquired using a Bruker spectrometer (FT-500 and 400 MHz) with tetramethylsilane (TMS) as the internal standard. The elemental analyses were done by the CHN-O elemental analyzer by GmbH-Germany for contents of C, H, and N (The results are reported within ±0.4% of the calculated values). The mass data were recorded on a LC-MS (Agilent 6410) at 70 eV. Merck silica gel 60 F254 plates were used for analytical thin-layer chromatography (TLC). Particle size 0.06-0.20 mm (Merck) was used for column chromatography.

### α-Bromo(4-chlorophenyl)acetic acid(1)

A solution of 4-chlorophenylacetic acid (10 g, 58.62 mmol), phosphorus trichloride (0.52 g, 4.16 mmol), and bromine (9.99 g, 62.43 mmol) was refluxed in 750 ml of benzene for three days. After cooling the solution to 25°C, it was filtered. Obtained brown oil by the solvent evaporation under reduced pressure was crystallized from ligroin. Finally, the crystals were filtered, washed by cool ligroin, and then dried.

Yield: 60%; mp = 98-100°C; IR (KBr, cm^-1^) ν_max_: 2400-3400(OH), 1720(C=O), 670 (C-Br). ^1^H NMR (CDCl_3_, 400 MHz): *δ* ppm 7.58 (d, 2H, J = 8.5 Hz, phenyl), 7.47 (d, 2H, J = 8.5 Hz, phenyl), 5.80 (s, 1H, CHBr).

### Methyl α-Bromophenylacetic acid (2)

α-Bromo(4-chlorophenyl) acetic acid (10g, 40.08mmol) obtained from the previous step and concentrated sulfuric acid (5 g) were refluxed in methanol (40 mL) for 4 hours. The solvent was evaporated by reduced pressure. Dichloromethane (50 mL) and sodium bicarbonate (NaHCO_3_) solution (50 mL) were added to the precipitate. After drying the dichloromethane phase by sodium sulfate (Na_2_SO_4_), the solvent was evaporated. Further, methyl ester product was achieved by distillation under reduced pressure.

Yield: 90%; IR (KBr, cm^-1^) ν_max_: 1750(C=O), 682(C-Br). ^1^H NMR (CDCl_3_, 400 MHz): *δ* ppm 7.49 (d, 2H, J = 8.52 Hz, phenyl), 7.34 (d, 2H, J = 8.52 Hz, phenyl), 5.32 (s, 1H, CHBr), 3.78 (s, 1H, ester CH_3_).

### Methyl 2-(4-chlorophenyl)-2-(4-(3-chlorophenyl)-3-oxopiperazin-1-yl)acetate (5)

1-(3-chlorophenyl) piperazin-2-one hydrochloride (5 g, 20.24 mmol) was added to methyl α-bromo (4-chlorophenyl)acetate (5.33 g, 20.24 mmol) in 50 ml of methanol along with sodium bicarbonate (3.4 g, 40.48 mmol) and the mixture was stirred at 80°C. After 6 hours, precipitated solids were separated by filtration, and the solvent was evaporated under reduced pressure. Then, the precipitate was dissolved in ethyl acetate (60 mL) and washed by water (30 mL). The separated organic phase washed with distilled water and cooled to -10°C. A mixture of 10 g of ice and 5 mL concentrated HCl was then added. Finally, the filtered precipitate was dried to obtain the expected product.

Yield: 69%; mp = 100-102°C; IR (KBr, cm^-1^) ν_max_: 3045(Aromatic), 1740 (ester C=O), 1675 (amide C=O), 1585 (aromatic C=C), 1290 (C-O). ^1^H NMR (CDCl_3_, 500 MHz): *δ* ppm 7.55-7.33 (m, 8H, phenyl), 4.99 (s, 1H, CHCOOMe), 3.80 (d, 2H, J=18.5 Hz, piperazinone), 3.68 (s, 3H, ester CH_3_), 3.36 (s, 2H, piperazinone), 3.15 (d, 2H, J=18.5 Hz, piperazinone). ^13^C NMR (CDCl_3_, 125 MHz,) *δ* ppm 169.20, 163.90, 142.81, 134.10, 132.86, 131.04, 130.46, 129.03, 126.61, 125.91, 124.39, 118.21, 68.75, 53.53, 52.64, 47.72, 46.77. MS (ESI): 394.39 [M+H]; Anal.

Calcd. for C_19_H_18_Cl_2_N_2_O_3_: C58.04, H 4.62, N 7.11; Found: C 57.91, H 4.62, N 7.15 %.

### 2-(4-Chlorophenyl)-2-(4-(3-chlorophenyl)-3-oxopiperazin-1-yl)acetic acid (6)

A solution of **5** (5.10g, 13 mmol) and NaOH (1.56 g, 39 mmol) was stirred in 50 mL MeOH/H_2_O (50:50) overnight at 25°C. After evaporation of the MeOH, the remaining suspension was adjusted with 200mL additional H_2_O and washed with Et_2_O (2×100 mL). HCl 1N (pH<3) was added to the residual solution, and extraction was done three times, each of 50 mL of EtOAc. The brine was used for washing the organic layer, and then it was dried by sodium sulfate (Na_2_SO_4_). The solvent evaporated under reduced pressure to obtain colorless oil.

Yield: 83%; mp = 219-220°C; IR (KBr, cm^-1^) ν_max_: 3300-2300(OH), 1740(Acid C=O), 1675(Amid C=O). ^1^H NMR (CDCl_3_, 500 MHz): δppm 7.60-7.33 (m, 8H, Phenyl), 5 (s, 1H, CHCOOH), 3.83 (brs, 2H, piperazinone), 3.35 (s, 2H, piperazinone), 3.27 (brs, 2H, piperazinone).^13^C NMR (CDCl_3_, 125 MHz,) *δ* ppm 169.57, 163.26, 142.68, 134.18, 132.88, 131.23, 130.51, 129.02, 126.73, 125.96, 124.45, 121, 69.06, 53.1, 47.25, 45.19. MS (ESI): 380.09 [M+H]; Anal. Calcd. for C_18_H_16_Cl_2_N_2_O_3_: C57.01, H 4.26, N 7.37; Found: C 56.89, H 4.24, N 7.41 %.

### General procedure for the synthesis of 7a-7c

2.3 mL of oxalyl chloride (26.5 mmol) was added dropwise to the solution of derivative **6** (5g, 13.19 mmol) in dry dichloromethane (50 mL) and DMF (0.5 mL) at 0^o^C over 30 minutes. The reaction was stirred 24 hours at 25°C. The dichloromethane was evaporated under vacuum to yield acyl chloride and used without any purification.

0.5 g of acyl halide (1.257 mmol) in dry dichloromethane (3 mL) was added dropwise to a stirred solution of appropriate amine (1.5 mmol) and 0.3 g of triethylamine (3 mmol) in dry dichloromethane (10 mL) at 0°C. The solution was stirred at 25°C for 3.5 hours, and the dichloromethane was evaporated under reduced pressure. The precipitate was dissolved in 10 mL EtOAc and washed with distilled water (3×10 mL). The EtOAc was dried by sodium sulfate (Na_2_SO_4_) and filtered. Then, the EtOAc was evaporated under reduced pressure. The purification of obtained products was done using column chromatography eluted with hexane/EtOAc. The yields of reactions ranged between 50 to 80%.

### 2-(4-Chlorophenyl)-2-[4-(3-chlorophenyl)-3-oxopiperazin-1-yl]-N-hydroxyacetamide (7a) 

Yield: 55%; mp = 260°C; IR (KBr, cm^-1^) ν_max_: 3300-2500(OH), 3200(NH), 1670(C=O), 1660(C=O). ^1^H NMR (DMSO-d_6_, 500 MHz): *δ* ppm 7.52-7.32 (m, 8H, phenyl), 3.92 (s, 1H, CHCONHOH), 3.48 (dt, 2H, J=33.25 Hz, J=5 Hz, piperazinone), 3.34 (s, 2H, piperazinone), 3.13 (dd, 2H, J=50 Hz, J=16.25 Hz, piperazinone).^13^C NMR (DMSO-d_6_, 125 MHz) *δ* ppm 166.1, 162.17, 143.75, 136.36, 133.28, 133, 128.8, 128.72, 126.7, 126.24, 124.64, 122.54, 69.57, 60.59, 55.8, 49.35. MS (ESI): 395.29 [M+H]; Anal. Calcd. for C_18_H_17_Cl_2_N_3_O_3_: C54.84, H 4.36, N 10.65; Found: C 54.66, H 4.34, N 10.69 %.

### 2-(4-Chlorophenyl)-2-[4-(3-chlorophenyl)-3-oxopiperazin-1-yl]acetamide(7b) 

Yield: 79%; mp = 254-256 ^o^C; IR (KBr, cm^-1^) ν_max_: 3380-3130(NH_2_), 1675(C=O), 1645(C=O). ^1^H NMR (DMSO-d_6_, 500 MHz): *δ* ppm 7.38-7.13 (m, 8H, phenyl), 4.54 (s, 1H, CHCONH), 3.66 (t, 2H, J=5.8 Hz, piperazinone), 3.46 (s, 2H, piperazinone), 3.04 (t, 2H, J=5.8 Hz, piperazinone). ^13^C NMR (DMSO-d_6_, 125 MHz) *δ* ppm 171.02, 162.77, 142.57, 136.89, 134.11, 132.76, 131.21, 130.74, 128.54, 123.4, 119.42, 119.11, 69.48, 55.33, 49.03. MS (ESI): 379.25 [M+H]; Anal. Calcd. for C_18_H_17_Cl_2_N_3_O_2_: C57.16, H 4.54, N 11.13; Found: C 56.99, H 4.54, N 11.09 %.

### 2-(4-Chlorophenyl)-2-[4-(3-chlorophenyl)-3-oxopiperazin-1-yl]acetohydrazide(7c) 

Yield: 66%; m.p = 256-258 ^o^C; IR (KBr, cm^-1^) ν_max_: 3270-3180 (NH_2_ and NH), 1680(C=O), 1651 (C=O). ^1^H NMR (DMSO-d_6_, 500 MHz): *δ* ppm 7.52-7.31 (m, 8H, phenyl), 3.92 (s, 1H, CHCONHNH_2_), 3.64 (d, 2H, J=16.35 Hz, piperazinone), 3.42 (s, 2H, piperazinone), 3.12 (d, 1H, J = 16.25 Hz, piperazinone). ^13^C NMR (DMSO-d_6_, 125 MHz) *δ* ppm 168.80, 166.18, 143.74, 136.11, 133.3, 133.09, 131.23, 130.80, 128.77, 126.71, 126.25, 124.62, 70.43, 55.78, 49.33, 47.53. MS (ESI): 394.19 [M+H]; Anal. Calcd. for C_18_H_18_Cl_2_N_4_O_2_: C54.97, H 4.62, N 14.26; Found: C 54.79, H 4.61, N 14.21 %.

### General procedure for the synthesis of 7d-7g

To stirred, dry, and 15 mL boiling acetonitrile solution of an appropriate amine (3.14 mmol) was added a solution of acyl halide (0.5g, 1.257 mmol) in 5 mL dry acetonitrile dropwise. The solution was refluxed for 3 hours. Then, the acetonitrile was evaporated under reduced pressure. The crude product was dissolved in EtOAc (15 mL) and washed using water (3×10 mL). The EtOAc was dried over sodium sulfate (Na_2_SO_4_) and evaporated. Finally, products were purified through recrystallization using EtOAc/n-hexane as the mobile phase.

### N-(Aminocarbonyl)-2-(4-chlorophenyl)-2-[4-(3-chlorophenyl)-3-oxopiperazin-1-yl]acetamide (7d)

Yield: 51%; mp = 282-284°C; IR (KBr, cm^-1^) ν_max_: 3375-3125(NH_2_, NH), 1680(C=O), 1651(C=O), 1635(C=O). ^1^H NMR (DMSO-d_6_, 500 MHz) *δ* ppm 7.49-7.30 (m, 8H, phenyl), 4.23 (s, 1H, CHCONH), 3.37 (d, 2H, J=11 Hz, piperazinone), 3.17 (s, 2H, piperazinone), 3.10 (d, 1H, J=11 Hz, piperazinone). ^13^C NMR (DMSO-d_6_, 125 MHz) *δ* ppm 173.13, 172.27, 166.78, 143.57, 138.99, 132.82, 131.18, 130.42, 130.34, 127.65, 126.09, 125.76, 124.13, 75.5, 55.81, 49.23, 47.35. MS (ESI): 421.99 [M+H]; Anal. Calcd. for C_19_H_18_Cl_2_N_4_O_3_: C54.17, H 4.30, N 13.30; Found: C 54.36, H 4.31, N 13.27 %.

### 1-(3-Chlorophenyl)-4-[1-(4-chlorophenyl)-2-(1H-imidazol-1-yl)-2-oxoethyl]piperazin-2-one (7e)

Yield: 35%; mp = 206-209°C; IR (KBr, cm^-1^) ν_max_: 1690 (C=O), 1650(C=O). ^1^H NMR (DMSO-d_6_, 400 MHz): *δ* ppm 7.85 (s, 1H, imidazole), 7.79-6.92 (m, 10H, phenyl and imidazole), 4.39 (s, 1H, CHCONH), 3.59 (d, 2H, 2H, J=9.2 Hz, piperazinone), 3.43 (s, 2H, piperazinone), 3.05 (d, 2H, J=9.2 Hz, piperazinone). ^13^C NMR (DMSO-d_6_, 75 MHz) *δ* ppm 165.15, 157.22, 150.41, 141.65, 136.91, 134.69, 134.36, 130.31, 130.24, 127.79, 126, 123.51, 121.11, 117.59, 51.14, 48.93, 48.57.MS (ESI): 430.29 [M+H]; Anal. Calcd. for C_21_H_18_Cl_2_N_4_O_2_: C58.75, H 4.24, N 13.06; Found: C 58.58, H 4.24, N 13.09 %.

### N-(aminocarbonothioyl)-2-(4-chlorophenyl)-2-[4-(3-chlorophenyl)-3-oxopiperazin-1-yl]acetamide (7f)

Yield: 63%; mp = 265-267°C; IR (KBr, cm^-1^) ν_max_: 3400-3250 (NH_2_, NH), 1680 (C=O), 1640 (C=O), 1480(C=S). ^1^H NMR (DMSO-d_6_, 400 MHz): *δ* ppm 7.54-7.33 (m, 8H, phenyl), 4.74 (s, 1H, CHCONH), 3.70 (brd, 2H, piperazinone), 3.51 (s, 2H, piperazinone), 3.33 (brd, 2H, piperazinone).^13^C NMR (DMSO-d_6_, 75 MHz) *δ* ppm 187.81, 165.72, 164.5, 142.49, 138.48, 135.64, 134.88, 133.84, 131.26, 128.76, 118.8, 117.72, 50.47, 49.16, 48.18, 47.45.MS (ESI): 438.69 [M+H]; Anal. Calcd. for C_19_H_18_Cl_2_N_4_O_2_S: C52.19, H 4.16, N 12.82; Found: C 52.11, H 4.14, N 12.83 %.

### N-[amino(imino)methyl]-2-(4-chlorophenyl)-2-[4-(3-chlorophenyl)-3-oxopiperazin-1-yl]acetamide (7g)

Yield: 33%; mp = 265-267 oC; IR (KBr, cm^-1^) ν_max_: 3400-3180 (NH_2_, NH), 1680 (C=O), 1640 (C=O), 1480(C=S). ^1^H NMR (DMSO-d_6_, 400 MHz): *δ* ppm 7.41-7.11 (m, 8H, phenyl), 4.14 (s, 1H, CHCONH), 4.02 (s, 2H, piperazinone), 3.71 (t, 2H, J=5.4 Hz, piperazinone), 3.63 (t, 2H, J=5.4 Hz, piperazinone).^13^C NMR (DMSO-d_6_, 125 MHz) *δ* ppm 166.72, 164.5, 139.59, 137.58, 136.64, 132.88, 130.24, 128.86, 128.76, 120.61, 118.52, 117.87, 52.77, 48.16, 47.48, 43.45.MS (ESI): 421.09 [M+H]; Anal. Calcd. for C_19_H_19_Cl_2_N_5_O_2_: C54.30, H 4.56, N 16.66; Found: C 54.14, H 4.55, N 16.63 %.

### Growth inhibition assay

Compounds **5, 6,**and **7a-7g** were evaluated forcytotoxic activity against HT29 (human colonic adenocarcinoma cells), A549 (adenocarcinoma human alveolar basal epithelial cells) as cancerous cell lines and one normal lung MRC-5 cells (normal fetal lung fibroblasts) using MTT assay. Seven concentrations (0.5-1000µM) of each compound were prepared. After seeding the cell suspensions (1 × 10^5^ cells/mL) in 96-well plates, they were incubated at 37°C to adhere to the cells. The cells were treated with mentioned synthesized compounds for 72 hours. Culture medium were removed and MTT [3-(4, 5-dimethylthiazol-2-yl)-2,5-diphenyl tetrazolium bromide] (25 μL, 4 mg/mL in PBS) were added to each well. After 3 hours of incubation at 37°C the medium was removed. By adding 100 μL of dimethyl sulfoxide per well and shaking for 15 min at 37°C, the purple formazan crystals were dissolved. The absorbance of wells was read at 570 nm using plate reader (sunrise Tecan, Austria). The results of each experiment which was done in triplicate in MTT assay were mentioned as mean ± SD.^19^

### Statistical analysis

The IC_50_ values were measured by GraphPad Prism v5.0.4.533 (GraphPad Software, San Diego, CA, USA).

## Results and Discussion

In the research, various novel piperazin-2-one derivatives, which contained guanidine, thiourea, hydrazide, and etc., were synthesized, characterized, and assayed against HT29, A549, and MRC-5 cell lines to study the relationship between structure and cytotoxicity.

**Scheme 1:**
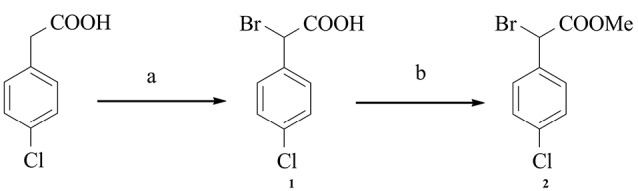
a) Br_2_, PCl_3_, benzene, reflux, 3 days; b) H_2_SO_4_, CH_3_OH, reflux, Overnight.^21^

Intermediate 2 was synthesized in a 50% yield via the reaction of 4-chlorophenylacetic acid with bromine and phosphorus trichloride in benzene. It was then esterified using a mixture of methanol with sulfuric acid as illustrated in scheme 1.^20,21^

**Scheme 2:**
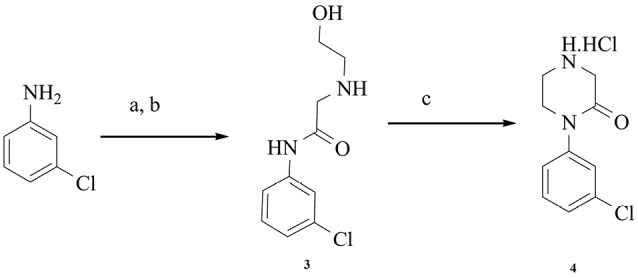
a) Chloroacetyl chloride, IpAc 0°C; b) Ethanolamine, IpAc, 55^o^C, 1 h; c) Tributylphosphine, DIAD, EtAc, -10°C.^22,23^

**Scheme 3:**
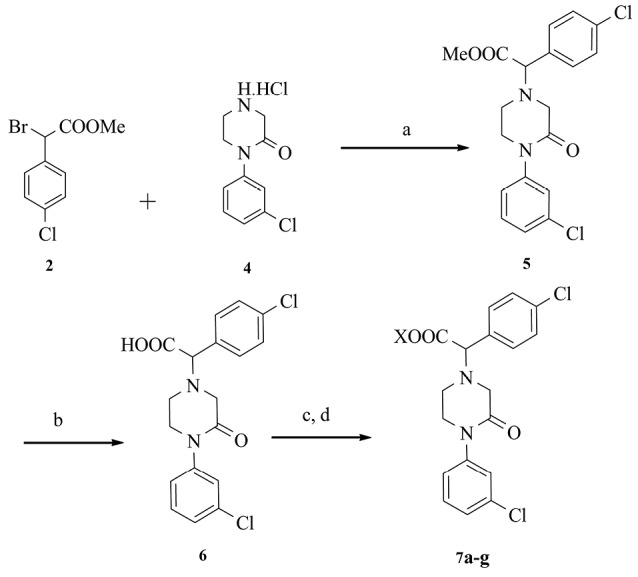
a) NaHCO_3_, MeOH, 80°C, 4 h; b) NaOH, MeOH/H_2_O, Overnight, r.t; c) Oxalyl chloride, CH_2_Cl_2_, DMF, 0°C; d)appropriate amine, CH_2_Cl_2_ or CH_3_CN.

Intermediate **4** and L-778,123 were synthesized according to well-known methods described previously.^21,22^ scheme 2 displays the synthetic route for intermediate **4**.

scheme 3 reveals the synthetic method for the preparation of the title compounds **5**, **6**, and **7a-g**.

The intermediate **5** was synthesized by the reaction of intermediates **2** and **4** using potassium carbonate (K_2_CO_3_) in methanol.^24^ The derivative **5** was hydrolyzed under basic conditions to **6** derivatives.^25^ The product **6** was converted to acyl halide by thionyl chloride in dichloromethane and dimethylformamide (DMF).^26^ The compounds **7a-g** were prepared from acyl halide and selected amine in dichloromethane or acetonitrile.^27,28^

Table 1 reports the results of cytotoxicity as IC_50_ (µM) of the compounds. The majority of final compounds indicated a significant cytotoxic activity on both cancer cell lines at concentrations <500 µM except for compounds **5** and **6**. The guanidine derivative (compound **7g**) exhibited the highest cytotoxicity against two investigated cancer cells at concentrations <2 µM, which were lower than doxorubicin on both cell lines. The other potent compounds were the thiourea and hydrazide derivatives (compounds **7f**and**7c**). The cytotoxic activity of compound **7e**with imidazole substituent did not change significantly compared to L-778,123. The compound **7d** showed significant cytotoxic effect, but it was less than L-778,123 (as the standard compound). The compounds **7a**and**7b** indicated cytotoxicity near 500 µM. The compounds **5** and **6**, however, did not indicate the significant cytotoxic effect on both cells (>1 μM).

**Table 1:** Cytotoxic activities (IC_50_, µM) of intermediate 5, 6 and compounds 7a-7g on A549 (lung cancer), HT-29 (colon cancer), and MRC-5 (normal fetal lung fibroblasts) cells

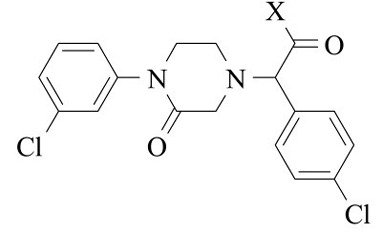				
**Compounds**	**X**	**A549**	**HT29**	**MRC-5**
**5**	-OMe	>1000	>1000	>1000
**6**	-OH	>1000	>1000	>1000
**7a**	-NHOH	431.67±2.85	498.17±3.73	>1000
**7b**	-NH_2_	317.44±2.92	398.18±1.47	>1000
**7c**	-NHNH_2_	22.67±0.83	49.28±0.68	125.71±1.26
**7d**	-NHCONH_2_	195.18±0.93	193.74±1.02	251.86±1.93
**7e**	Imidazole	110±2.51	128.99±2.86	194.23±1.44
**7f**	-NHCSNH_2_	5.11±1.41	6.21±1.21	8.75±0.53
**7g**	-NHCNHNH_2_	1.3±0.22	1.8±0.13	5.28±0.41
**L-778,123**		101±2	125±2	150.70±1.72
**Doxorubicin**		4.1±0.1	3±0.1	0.85±0.12

Compounds **5**, **6**, **7a**, and **7b** did not show cytotoxicity against MRC-5. Compounds**7f** and **7g** showed significant cytotoxicity against normal MRC-5 cells. All compounds were significantly more cytotoxic toward both cancer cell lines as compared to normal MRC-5 cells.

The cytotoxic mechanism of these derivatives is not clear. Although it has been suggested that they exert their effects through inhibiting FTase, most synthesized derivatives which had not indicated acceptable cytotoxicity have been effective FTIs and vice versa.^13-16,18^

The benzyl group of the derivatives can be substituted by different electron-withdrawing groups like nitrile- or chloro- moieties at the para position. Several FTIs such as tipifarnib have chloro group instead of cyano group at this position. These FTIs have indicated higher cytotoxicity than L-778,123. We predicted that the substitution of 4-CN for 4-Cl could improve the cytotoxic activity of the compounds. So, 4-cyanobenzyl was substituted for 4-chlorobenzyl in these derivatives.^17,22,23,26,29-31^

Lung and colon cancers were selected for cytotoxic activity assessment because they are the most important kinds of cancers causing death over the world according to WHO reports. Besides, HT-29 (colon cancer) and A549 (lung cancer) cells are routine cells in cytotoxicity evaluation in many studies. Also, the cell lines showed wild-type K-ras.^32,33^

The terminal aliphatic nitrogen atoms of bioisosteres may have an essential effect in cytotoxicity. The cytotoxicity of compound **7e** showed that only bioisosteric replacement significantly changes the cytotoxic effect. Replacement of imidazole with guanidine, thiourea, and hydrazide (compounds **7g**, **7f**, and **7c**) led to significantly better cytotoxic effect on all three cell lines in comparison with L-778,123. The improvement in the cytotoxic effect may be as a result of the fact that terminal atoms have higher electron density.^34-36^ It has been demonstrated that bioisosteres of imidazole show cytotoxicity through different mechanisms like inhibition of inosine monophosphate dehydrogenase by guanidine-based compounds. The cytotoxic effect of potent compounds (**7c**, **7f**, and **7g**) can be related only to the cytotoxicity of imidazole bioisosteres.^16,37-39^ Other substitutes did not significantly increase the cytotoxic effect in comparison with the above mentioned potent compounds. It can be attributed to the amide groups with lower electron density. Compound **7b** indicated better cytotoxic activity than compound **7a.** It seems that the substitution of hydroxyl on the amine group can decrease the electron density on terminal nitrogen. Finally, it seems rearrangement of groups based on tipifarnib structure can increase the cytotoxicity against three cell lines. The IC_50_ of the guanidine derivative in this study showed significant decrease against all cell lines in comparison with previous work.^25^

Nevertheless, for understanding the precise mechanisms of the action of the synthesized compounds, further studies are needed.

## Conclusion

A set of bioisosteres of imidazole containing-derivatives 1-(3-chlorophenyl)piperazin-2-one group were prepared, characterized by various methods including IR, ^1^HNMR, ^13^CNMR, and Mass spectroscopy, and tested for cytotoxic activity. The piperazinone derivatives with guanidine substituent (**7g**) showed the highest potency against all cell lines. Notably, it showed better cytotoxicity than doxorubicin against both cancer cell lines. So it can be a proper lead compound to design novel anticancer compounds. The electron density of terminal atoms of bioisosteres may be an essential factor in the cytotoxic activity. Finally, rearrangement of groups similar to tipifarnib structure can significantly enhance the cytotoxicity. All synthetic compounds showed lower cytotoxicity against healthy cell lines than cancer cell lines.

## Ethical Issues

Not applicable.

## Conflict of Interest

The authors declare no conflict of interest in this article’s content.

## Acknowledgments

The faculty of pharmacy and the tuberculosis and lung disease research center, Tabriz University of medical sciences, have supported this work financially.
